# Changes in the pattern of plasma extracellular vesicles after severe trauma

**DOI:** 10.1371/journal.pone.0183640

**Published:** 2017-08-24

**Authors:** Sahithi J. Kuravi, Clara M. Yates, Mark Foster, Paul Harrison, Jon Hazeldine, Peter Hampson, Chris Watson, Antonio Belli, Mark Midwinter, Gerard B. Nash

**Affiliations:** 1 Institute of Cardiovascular Sciences, College of Medical and Dental Sciences, University of Birmingham, Birmingham, United Kingdom; 2 NIHR Surgical Reconstruction and Microbiology Research Centre, University Hospitals Birmingham NHS Foundation Trust, Birmingham, United Kingdom; 3 Institute of Inflammation and Ageing, College of Medical and Dental Sciences, University of Birmingham, Birmingham, United Kingdom; 4 Department of Haematology, Queen Elizabeth Hospital, Birmingham, United Kingdom; Institut d'Investigacions Biomediques de Barcelona, SPAIN

## Abstract

**Background:**

Extracellular vesicles (EV) released into the circulation after traumatic injury may influence complications. We thus evaluated the numbers of EV in plasma over 28 days after trauma and evaluated their pro-coagulant and inflammatory effects.

**Methods and findings:**

37 patients suffering trauma with an injury severity score >15 were studied along with 24 healthy controls. Plasma samples were isolated by double centrifugation (2000*g* 20min; 13000*g* 2min) from blood collected from within an hour up to 28 days after injury. Plasma EV were counted and sized using nanoparticle tracking analysis (NTA); counts and cellular origins were also determined by flow cytometry (FC) using cell-specific markers. Functional effects were tested in a procoagulant phospholipid assay and in flow-based, leukocyte adhesion assay after endothelial cells (EC) were treated with EV.

We found that EV concentrations measured by NTA were significantly increased in trauma patients compared to healthy controls, and remained elevated over days. In addition, or FC showed that patients with trauma had higher numbers of EV derived from platelets (CD41+), leukocytes (CD45+) and endothelial EC (CD144+). The increases were evident throughout the 28-day follow-up. However, the FC count represented <1% of the count detected by NTA, and only 1–2% of EV identified using NTA had a diameter >400nm. The procoagulant phospholipid activity assay showed that patient plasma accelerated coagulation on day 1 and day 3 after trauma, with coagulation times correlated with EV counts. Furthermore, treatment of EC for 24 hours with plasma containing EV tended to increase the recruitment of peripheral flowing blood mononuclear cells.

**Conclusions:**

EV counted by FC represent a small sub-population of the total load detected by NTA. Both methods however indicate a significant increase in plasma EV after severe traumatic injury that have pro-coagulant and pro-inflammatory effects that may influence outcomes.

## Introduction

Systemic complications following severe traumatic injury can lead to late morbidity and mortality [1, 2]. For instance, persistent local and systemic release of proinflammatory mediators can cause the systemic inflammatory response syndrome [3–5]. Along with increased release of soluble mediators such as cytokines, elevated numbers of extracellular vesicles have been reported in circulating blood after severe trauma [6, 7]. Whether the levels or bioactivity of these vesicles influence the inflammatory complications of traumatic injury remains uncertain.

Extracellular vesicles (EV) are membrane fragments of diameter ~0.05 to 1μm, released from cells upon activation or when undergoing apoptosis, and previoulsy termed microparticles; they include exosomes (of intracellular origin, <0.1μm) and ectosomes or microvesicles shed from the plasma membrane (~0.1 to 1μm) (8). EV contain membrane proteins, mRNA, miRNA, cytokines and chemokines [9,10]. They may be vectors of intercellular exchange with significant roles in coagulation, inflammation, immune responses, angiogenesis and cancer [11–13]. EV released from endothelial cells (EC) and blood cells circulate in the peripheral blood with platelet EV typically forming a large proportion [14–17]. Increases in circulating EV have been associated with procoagulant and inflammatory conditions in a variety of clinical disorders [18, 19] and may have a role in initiation or progression of secondary inflammatory complications [6, 13, 20, 21].

Previous studies have characterized EV in blood collected after traumatic injury, demonstrating their levels, cellular origins, adhesion molecule expression and procoagulant activity [6, 7, 16, 22, 23]. Higher levels of platelet microparticles and platelet-leukocyte aggregates were detected in blood of patients with trauma and sepsis compared to healthy controls [6]. However, these studies did not follow individual patients over prolonged periods, to see how responses evolved. Furthermore, previous trauma studies have not compared different methods for detecting the EV. These may be based on functional effects, such as procoagulant activity, or on direct counting, most often by flow cytometry (FC; which can only detect larger vesicles of hundreds of nanometers), but also by nanoparticle tracking analysis (NTA; which detects vesicles down to tens of nanometers) [7, 16]. However, a number of studies have pointed out the limitations of the methods for detecting or quantifying EV which have a wide range of sizes [8, 24]. These studies demonstrated great differences in the plasma EV count between methods (ranging between 10^4^ and 10^12^/ml) and recommended the use of a combination of assays in clinical studies.

Based on the questions raised above, we set out to analyse the total EV load (microvesicles and exosomes, potentially including apoptotic bodies) in blood of patients suffering severe traumatic injury, with follow up from within hours up to 28 days. We used NTA and FC, the former allowing a fuller count of total load and evaluation of the size distribution, the latter allowing analysis of the cellular origin of larger vesicles. Given the inflammatory and coagulation responses observed after trauma [7,25], we evaluated pro-coagulant and pro-inflammatory activity in clotting time and leukocyte adhesion assays. Thus, this is the first longitudinal study to quantify EV release using multiple methodologies and to carry out parallel functional analyses.

## Methods

### Subjects, blood collection, isolation of plasma EV and study design

Blood samples were collected from 37 patients who had suffered severe trauma with an injury severity score >15. Blood was also collected from 24 healthy adult volunteers of similar age, after informed consent. The patients were part of either: (i) the Steroids and Immunity from Injury through to Rehabilitation Study (SIRS; n = 19 between May 2012—March 2013) in which blood was drawn 6 hours to 24 hours after injury (day 1) and then at intervals over a period up to 28 days; (ii) the Golden hour study (GHS; n = 18 between June 2014—July 2015) in which blood was withdrawn within 60 minutes of injury, later on day1 (6 hours to 24 hours after injury), and on day 3. At the time of first withdrawal of blood in the Golden Hour study, no treatments had started. By the time of the day 1 sample in either study, most patients had received crystalloid and over one half received blood or plasma. Moreover, about half were ventilated, and this could extend for up to 20 days. None of the patients included in this subset of these larger clinical studies received steroids.

The studies were performed in collaboration between the University of Birmingham, University Hospitals Birmingham NHS Foundation Trust and the Defence Medical Services (Royal Centre for Defence Medicine and Institute of Naval Medicine), after ethical approval by the National Research Ethics Service (approval numbers 11/SW/0177 and 13/WA/0399). All patients were consented, under guidance of the mental health capacity act, in accordance with the Declaration of Helsinki.

Blood was drawn into 3.2% citrate BD vacutainer. No donors were fasting, and venous blood was drawn either by venepuncture or in some patients, via intravenous cannula. Samples from SIRS patients were held on ice for up to 4 hours before processing to isolate EV could be carried out. Samples from GHS or controls were available within 1 hour and held at room temperature until processed. A number of studies have shown that cold storage causes changes in platelet shape suggesting a form of activation, and that there are deleterious effects of storing platelet isolates in the cold before transfusion [e.g., 26, 27]. Isolated platelets stored for days in the cold did yield slightly higher number of vesicles than platelets stored at room temperature (by about 50%; [28]). However, we are not aware of any studies of the effects cold exposure on EV number in blood. On the other hand, separate studies show that storage of blood at room temperature causes gradual increase in platelet-derived plasma EV over hours [29,30]. Thus we held blood available within an hour at room temperature, but for longer periods used cold storage, as noted above. The method of blood withdrawal itself has been shown to have little effect on EV number [30].

Our purpose was to prepare and evaluate function of samples representing the total load of EV in the plasma without cell contamination, rather than to isolate specific types or subsets of EV. We thus used a double centrifugation method similar to that recommended by the International Society for Thrombosis and Hemostasis for analysing plasma microparticles [31] and used in previous studies for this purpose [7,10]. Platelet free plasma, containing EV, was generated from the blood by centrifugation, initially at 2000g for 20min to yield platelet-poor plasma. This was centrifuged at 13000g for 2min to yield the EV supernatant. The plasma EV samples were stored in 100μl aliquots at -80°C until analysis. Previous studies have analysed effect of cold storage and have found minor changes in vesicle count if blood is double-centrifuged before freezing (and thus free of residual platelets) [29,30,32], but possible decrease with prolonged storage [29]. Cold storage was necessary to allow a range of analyses to be done on equivalent samples, and all samples for patients or controls were handled similarly.

Not all samples were subject to all analyses due to the volumes of samples available; numbers used for each analysis are shown in the Results. Samples from SIRS were used in all analyses, but samples from GHS were only sufficient for use in the analysis of total plasma EV number, analysed by nanoparticle tracking analysis (see below) which we considered the primary endpoint here, and which had the most discriminatory power based on sample number. Thus, the earliest sample tested (T0; within 60min) was from GHS only, day 1 and day 3 samples were available from GHS and SIRS, and later samples were from SIRS only.

### Determination of number and size of EV using nanoparticle tracking analysis (NTA)

Size distribution and EV concentration in plasma samples were determined using a Nanosight LM10 (Malvern Instruments, UK) equipped with NTA software 2.2 as described [33]. The minimum size detected was ~50nm. Plasma samples were diluted with filtered phosphate buffered saline (PBS; Sigma) to achieve optimum particle concentration of 10^8^−10^9^/ml. 300μL of sample was introduced into a chamber held on a light microscope, which was illuminated by laser at an angle to the optical axis of the microscope. A digital camera attached to the microscope visualized the scattered light from particles and images were captured at a rate of 30/s for 60 seconds. Individual particles were counted and tracked, and their Brownian motion analyzed to yield their velocity and hence diameter. Recording thus gave the frequency distribution of vesicle sizes and an estimate of the total number of EV/ml.

### Characterization of EV using FC

Plasma EV were labeled with cell-specific fluorescent antibodies: FITC-conjugated antibodies against CD45 (clone MEM-28, Immunotools) or CD41 (Clone 5B12, DAKO); APC-conjugated antibodies against CD42b (clone HIP1, BD Pharmingen) or CD144 (clone 16B1, ebioscience). 40μl plasma was incubated with an appropriate antibody or an isotype-matched control for 20mins at room temperature in the dark. Following incubation, samples were diluted to 200μl with filtered PBS containing 0.15% bovine serum albumin (PBSA) and analyzed on a BD Accuri C6 flow cytometer (BD, Oxford, UK).

**Fig 1:**
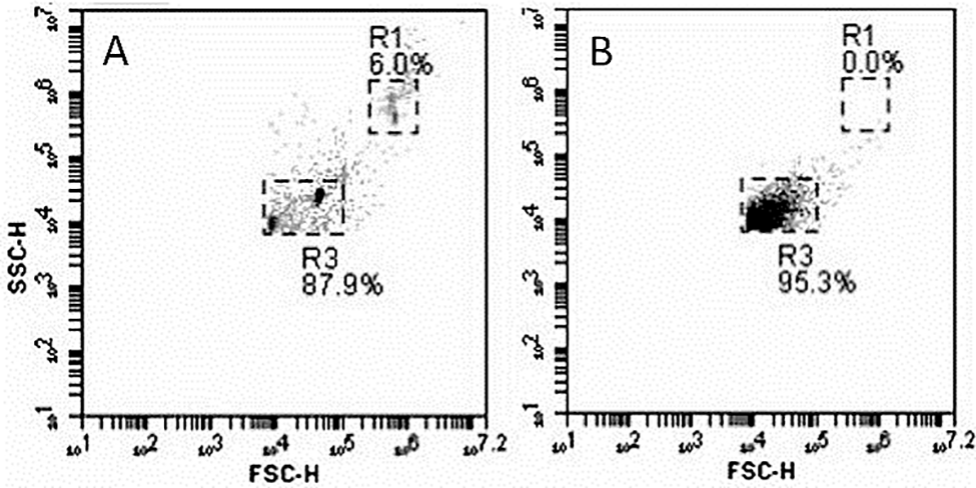
Gates used for counting EV by flow cytometry. A. Side-scatter vs. forward scatter plot for megamix fluorescent polystyrene beads of sizes 500nm, 900nm and 3μm. R3 defines the gate used for counting EV. R1 incorporates 3μm beads and the approximate position of intact platelets. B. Side-scatter vs. forward scatter plot for plasma extracellular vesicles. Approximately 95% of detected particles with side scatter and forward scatter greater than the lower limits set by R3, fell within R3.

The gating window for counting EV and discriminating against background noise was set using forward and side scatter plots for Megamix fluorescent polystyrene beads (BioCytex, France) of diameters 500nm, 900nm and 3μm. EV counts were taken from the gate that included 500nm and 900nm megamix beads (as shown in Fig 1). Extending this gate to include smaller particles was not possible, as noise detection in particle-free diluent became significant. Thus, we only analysed larger EV with this method, and relied on NTA to obtain data on numbers of smaller vesicles. The gate settings were maintained for measuring all the patient and control plasma samples.

Samples were analyzed at a low flow rate (14μl/min) until 2000 positive events were collected in the EV gate. The absolute EV numbers were acquired from counts in the microvesicle gate defined as above, for a known sample volume. The cell specific EV counts were obtained from fluorescent signals for the different cell markers analyzed one at a time, for particles that fell in light scatter gate and were positively labeled compared to fluorescent isotype controls.

### Procoagulant phospholipid (PPL) assay

An automated clotting time assay was performed (STA®-Procoag-PPL assay; Stago, Asnieres, France) to measure total procoagulant phospholipid in patient plasma compared against control plasma. The PPL assay is based on the anionic phospholipid-dependent activation of prothrombin by the factor Xa/factor Va complex. The clotting times derived from the assay are proportional to the quantity of PPL present in the sample. The test was run according to manufactures instructions. Briefly, 25 μl of test plasma was added to 25 μl of phospholipid-depleted, citrated, human plasma and incubated at 37°C. Coagulation was triggered by adding 100 μL bovine Factor Xa/CaCl2 reagent and clot formation was measured by the ACL Top analyzer (Werfen Diagnostics, Warrington, UK).

### Isolation of HUVEC, neutrophils and PBMC

Human umbilical vein EC (HUVEC) were isolated from umbilical cords by collagenase treatment as described previously [34]. Cords were obtained from the Human Biomaterials Resource Centre (University of Birmingham) after informed maternal consent. The cells were cultured at 37°C with 5% CO_2_ in M199 supplemented with 20% fetal calf serum, 10 ng/mL epidermal growth factor, 35 μg/mL gentamicin, 1 μg/mL hydrocortisone (Sigma-Aldrich, U.K.), and 2.5 μg/mL amphotericin B (Life Technologies, CA).

Blood was collected from healthy adult volunteers into EDTA tubes (Sarstedt Ltd., Leicester, U.K.) after informed consent. Neutrophils and peripheral blood mononuclear cells (PBMC) were isolated by overlaying blood on histopaque density gradients of histopaque 1077 over histopaque 1119 (Sigma) as described [35]. Collected cells were washed using PBSA and counted using a Cellometer auto T4 cell counter (Nexcelom) and diluted to 1x10^6^ cells/ml in PBSA.

### Leukocyte recruitment under flow

Primary HUVEC were subcultured into ibidi μ-Slide VI^0.4^ (Ibidi, Germany) for 24h until confluent. Confluent cultures were treated for 24h with patient or control plasma EV mixed 1:1 with culture medium including 5U/ml heparin to maintain anticoagulation, with 0 or 5 U/ml tumour necrosis factor-α (TNF) added for the last 4h. A flow-based adhesion assay was performed as described previously [36]. Briefly, ibidi slides were mounted on the stage of a phase-contrast video microscope enclosed in a 37°C perspex chamber. One end of the slide was connected to a Harvard syringe pump set to a flow rate equivalent to a wall shear stress of 0.05Pa. Leukocyte suspension was perfused over EC for 4min followed by washout with PBSA. After 5min washout, a series of microscopic fields were recorded along the centerline of the microslide and digitized images were analyzed offline using Image Pro software (Image-ProPlus). The numbers of adherent leukocytes were counted from the images and converted to leukocytes/mm^2^/10^6^ perfused based on the known field size, sample concentration and flow rate. Data for patient samples of EV were normalized to values obtained with control samples of EV, measured on the same day with the same HUVEC and leukocyte donors.

### Statistical analysis

Data were analyzed using GraphPad Priosm software (GraphPad Software Inc., LaJolla CA). Data were analyzed for normal distribution by D’Augustino-Pearson normality test. Results are expressed either as the mean ± standard error of the mean (SEM) or median with interquartile range for data that were or were not normally distributed. The variations in parameters between different treatments or times were analysed by analysis of variance for data that were normally distributed or by Friedman test (two-way) or Kruskal-Wallis (one-way) test or for data that were not normally distributed, with post-hoc Dunn test for comparison of pateints to control where appropraite. Correlation between counts from NTA and FC, between FC counts and blood counts for platelets and leukocytes, and between PPL and EV counts were tested using linear regression.

## Results

The demographic and clinical characteristics of the patients are shown in Table 1. Healthy controls had median age 34.5 years (range 24–43 years).

**Table 1:** Characteristics of patients.

Patient Characteristic	SIRS	Golden Hour
Number	19	18
Age (Years)	46 (18–84)	40 (19–82)
Brain Injury	5	11
Extracranial injury	4	16
Injury severity score	26 ± 5	23 ± 3
Glasgow coma scale (GCS)	14 (3–15)	12 (3–15)

Data are mean ± SEM, except age and GCS which are median (min—max).

### Number and size distribution of plasma EV analysed by NTA

**Fig 2:**
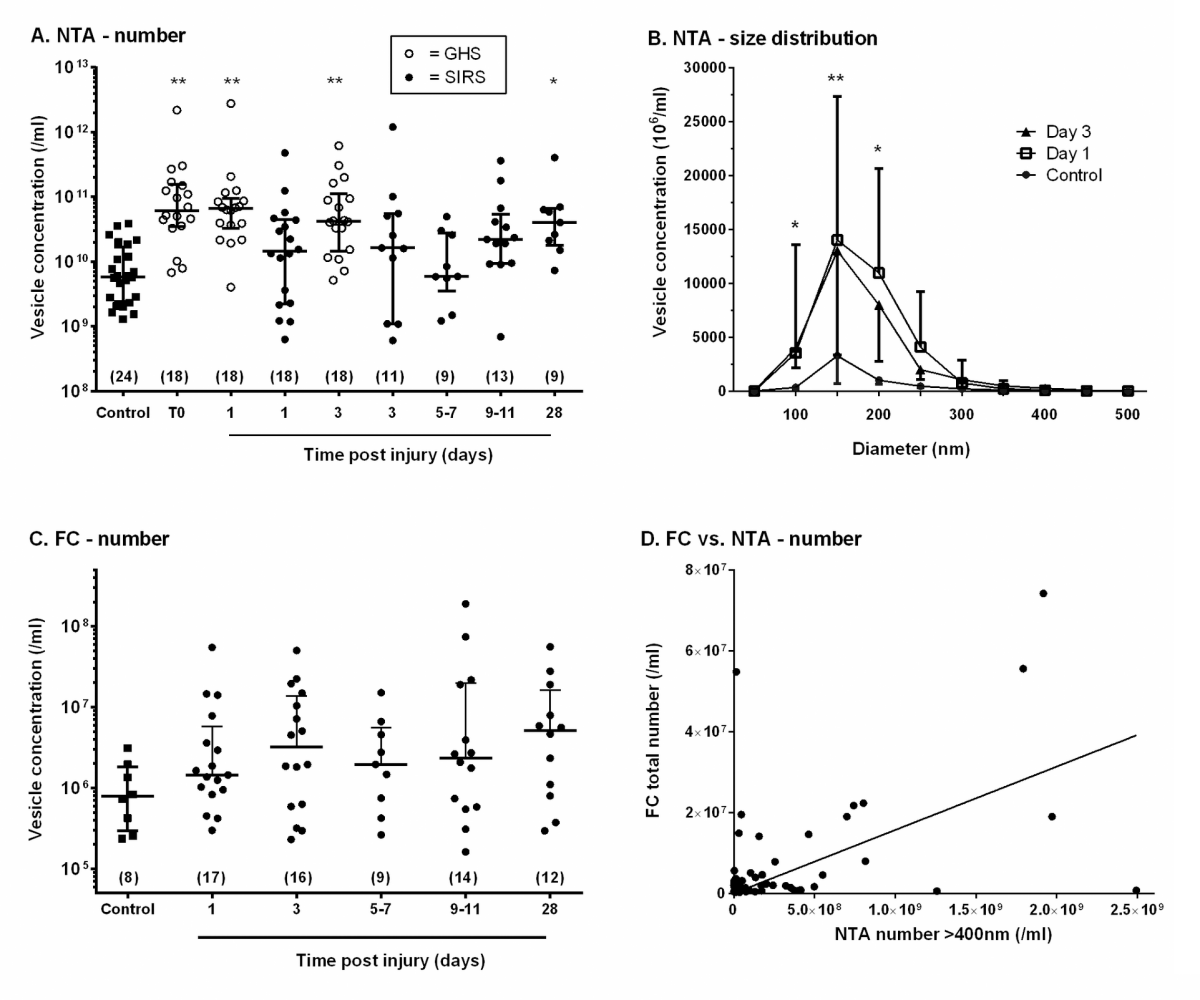
Concentrations and size distributions of EV in plasma of healthy controls and patients analysed by nanoparticle tracking analysis (NTA) and flow cytometry (FC). A. NTA analysis of the concentration of EV in plasma for controls and patients from GHS (○) or SIRS (●) at different times after injury. The data are shown as median ± inter-quartile range (number of donors in brackets). Kruskal-Wallis test showed significant variation between samples (p<0.01); * = p<0.05, ** = p<0.01 compared to control by post-hoc Dunn test. B. The frequency distribution of EV diameter derived by NTA in plasma from controls and patients at different times after injury. Data are for controls (n = 13), Day 1 (pooled GHS and SIRS, n = 36) and Day 3 (pooled GHS and SIRS, n = 27). Friedman test showed significant variation with particle diameter and subject group both (p<0.01). Subsequent Kruskal-Wallis test for each particle diameter class showed signficant variation between subject groups for 50–100, 100–150 and 150-200nm; * = p<0.05, ** = p<0.01 for Day 1 and for Day 3 compared to control by post hoc Dunn test. C. Flow cytometry analysis of the concentration of EV in plasma for controls and patients at different times after injury. The data are shown as median ± inter-quartile range (number of donors in brackets). D. Correlation of EV concentration measured by FC with EV concentration at diameter >400nm measured by NTA. Linear regression showed a significant correlation between FC and NTA concentrations (R^2^ = 0.24; P<0.01).

The concentration of EV in plasma and their size distribution were analysed by NTA as a function of time after traumatic injury, and compared to values for healthy controls. For the GHS patients, in the first hour after trauma, the EV concentration was approximately ten-fold higher than controls (Fig 2A). For the SIRS patients, the EV concentration was elevated over a prolonged period from day 1 onward and there was no significant correlation between number of vesicles and time. However, numbers of EV were not significantly greater than controls for individual days except day 28 (Fig 2A). The EV count was higher for the GHS patients overall than for SIRS, although the range was similar. Fig 2B shows the frequency distribution of particle diameters collected in 50nm intervals for controls versus day 1 and day 3 data, pooled for the two clinical studies (SIRS and GHS). Supplementary S1 Fig shows shows the frequency distributions for all days analysed, but without error bars for clarity. For all samples, the modal diameter was 100-150nm, where again, the count was higher for day 1 and day 3 post-injury than controls. There were only minor variations in the shape of the distribution, and in all cases there was only a small proportion of vesicles with diameter above 400nm. Since vesicles of this size should be detected by FC, their number was also quantified from the NTA data (Table 2). Between 1–2% of vesicles had diameter >400nm, but the proportion did not vary significantly between patients and controls, or with time after trauma.

**Table 2:** Percentage of extracellular vesicles with diameter >400nm detected by NTA.

Percentage of microvesicles with diameter >400nm						
Controls	Patients—days post trauma					
	T0	Day 1	Day 3	Day 5–7	Day 9–11	Day 28
1.5 ± 0.5 (13)	1.1±0.3 (18)	1.4 ± 0.5 (36)	2.5 ± 0.9 (29)	1.4 ± 0.6 (9)	2.4 ± 0.8 (13)	1.2 ± 0.4 (9)

The size distribution of plasma EV was analysed by NTA, and the percentage with diameter >400nm calculated. Data are mean ± SEM from (n) patients or controls. T0 refers to patients studied in the first hour after injury.

### Number of plasma EV analysed by FC and comparison to NTA

Flow cytometry detected EV that fell within a light scatter gate incorporating 500 and 900nm beads. Total counts were less than 1% of those measured by NTA, but again, plasma EV concentrations tended to be greater in trauma patients compared to healthy controls, and the values remained elevated throughout the study period (Fig 2C). However, the increase over control was not statistically overall. Golden-hour samples were not analysed by FC. Keeping in mind the different size ranges detectable by FC and NTA, we tested whether there was a correlation between numbers measured by FC and numbers >400nm measured by NTA. Fig 2D shows that the FC counts did correlate significantly with the number of larger vesicles counted by NTA. In contrast, the FC count did not correlate with numbers of smaller vesicles (<400nm) measured by NTA (not shown).

### Cellular origin of plasma EV analysed by FC

**Fig 3:**
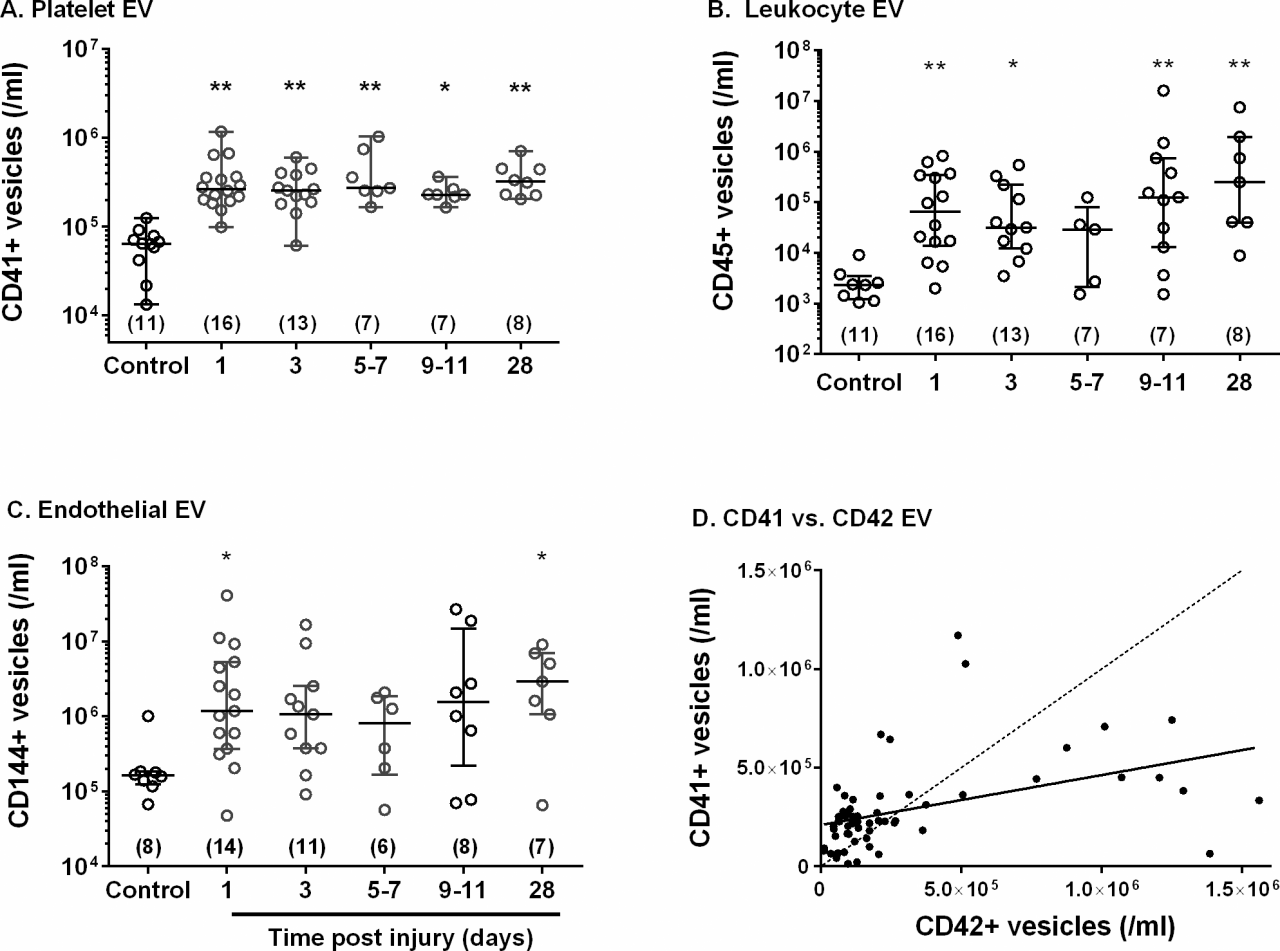
Concentrations of EV from different cellular origins in plasma of healthy controls and patients analysed by flow cytometry. EV in plasma from patients or controls were labelled with antibodies recognising markers for A. platelets (CD41^+^), B. leukocytes (CD45^+^) or C. endothelial cells (CD144^+^) and analysed by flow cytometry. In each case, Kruskal-Wallis test showed significant variation between samples but not with time post injury for samples from patients alone; * = p<0.05, ** = p<0.01 compared to control by post hoc Dunn test. D. Plot of numbers of CD41^+^ vs. CD42^+^ EV counts for all samples. Linear regression showed a significant correlation (R^2^ = 0.1909, p<0.01) with equation: CD41^+^ = 2.1x10^5^ + 0.25 CD42b^+^.

We analyzed the origins of circulating EV in plasma of trauma patients using cell specific markers for: platelets (CD41^+^); EC (CD144^+^); leukocytes (CD45^+^). Fig 3 shows that there were higher concentrations of each type of EV in the patient’s plasma on day1 when compared to controls. The numbers of specific EV again remained elevated over the 28 days. In addition, we compared the plasma levels of EV for platelet markers CD41^+^ or CD42b^+^. The number of CD42b^+^ EV (i.e., bearing GpIb) was on average similar to number of CD41^+^ EV (i.e., bearing GpIIbIIa). However, the correlation between the counts was weak (Fig 3D), with the CD41^+^ count relatively little influenced by the CD42b^+^ count. The coefficient of variation in CD42b^+^ counts was greater than for CD41^+^ counts overall, but CD42b counts for day 1 trauma patients were still statistically significantly elevated above controls (p<0.05 by Mann-Whitney U-test).

We also analysed the correlation between the number of cell-specific EV (using CD41^+^ for platelet-derived EV) and the number of circulating cells in the blood for platelets and leukocytes. None of the specific EV showed significant correlation with the respective cell counts (data not shown), indicating that variation in EV did not simply reflect differences in the numbers of cells in the circulation.

### Effect of plasma containing EV on coagulation and adhesion of flowing leukocytes

We evaluated the potential functional effects of EV in plasma. In the procoagulant PPL assay, plasma of trauma patients accelerated coagulation compared to control plasma. Coagulation times were: 80.2 ± 15.0s (mean ± SEM, n = 15) on day1 and 73.6sec ±1 5.9 (n = 12) on day 3 after injury, versus 106.2sec ± 6.6 (n = 8) for controls (p<0.01 for comparison of days 1 or 3 to controls by t-test). The data showed little overlap between post-trauma and control, suggesting high levels of procoagulant vesicles that could promote hypercoagulability in the patients. Interestingly, the clotting time was inversely correlated with the EV count of large vesicles obtained by flow cytometry (p<0.01 by linear regression); for the NTA total count the correlation was positive with borderline statistical significance (p = 0.054).

**Fig 4:**
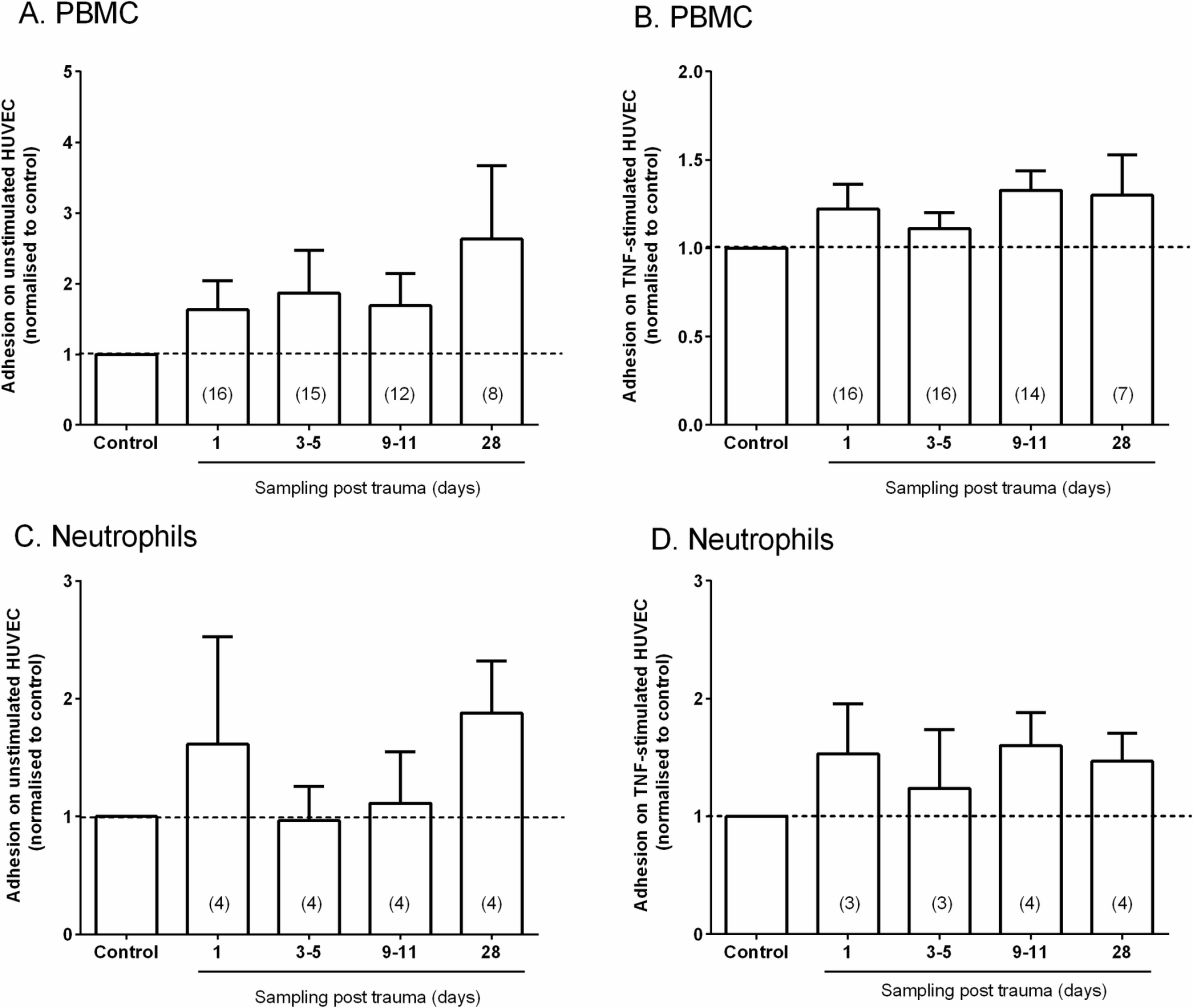
Effects of plasma containing EV on leukocyte adhesion to endothelial cells from flow. HUVEC was pre-treated for 24h with plasma EV from healthy controls or trauma patients, with or without treatment with TNF (5U/ml) for the last 4h. Peripheral blood mononuclear cells (PBMC) or neutrophils (PMN) were perfused at a wall shear stress of 0.05 Pa for 4min followed by 5min washout, and adherent cells counted. Results for patient plasma were expressed relative to healthy control plasma tested on the same day with the same HUVEC and leukocytes. A, B. PBMC adhesion after the absence or presence TNF. C, D. Neutrophil adhesion after the absence or presence TNF. All data are mean ± SEM (number of patients tested at each time in brackets). In A, the ratio of adhesion for patient:control plasma was significantly >1 for all samples tested (p<0.01), but the increase was not statistically significant at any particular day.

We also analysed the pro-inflammatory potential of EV by pre-treating EC with plasma containing EV for 24h, and testing the ability of EC to recruit flowing peripheral blood mononuclear cells (PBMC) or neutrophils. Treatment with plasma was carried out along with a low dose of TNF (5U/ml) for 4h or for otherwise stimulated EC, to test whether plasma contents including EV augmented a response to the cytokine or induced adhesion themselves. Plasma from trauma patients at day 1–28 showed generally higher but very variable adhesion of PBMC to an otherwise unstimulated EC compared to control plasma (Fig 4A). Overall, the ratio of adhesion for patient:control plasma was 1.8 ± 0.3; mean ± SEM from 55 comparisons to controls on different days (p<0.01), but the increase was not statistically significant at any particular day. With TNF stimulation, the effect of plasma treatment on adhesion of PBMC was less (overall ratio 1.2) and not statistically significant (Fig 4B). In the case of neutrophil adhesion, the highly variable effects of plasma from patients were not significant for unstimulated or TNF-treated EC (Fig 4C and 4D).

## Discussion

In this longitudinal study, we followed responses in patients for up to 28 days post severe traumatic injury, measuring numbers of plasma EV, identifying their cellular sources and analyzing the pro-coagulant and pro-inflammatory potential of these EV. The EV were analyzed by flow cytometry (measuring only larger vesicles, but allowing analysis of cellular origin) and by NTA (counting and measuring the size distribution of EV over a wide range). Both methods indicated that the number of circulating plasma EV was elevated significantly within the first day after injury compared to healthy controls. Perhaps surprisingly, values remained high thereafter for a prolonged period. There was a high degree of variability between samples and no significant effect of time after injury on the numbers was detected. Analysis of the sources of EV showed that elevated numbers could be attributed to release from circulating platelet and leukocytes and from EC, suggesting a widespread 'activation' of the vascular system. Examining the NTA data, there was no major shift in the size distribution of EV after trauma. Nevertheless, the number of the largest vesicles (>400nm), correlated with the number of EV detected by FC, indicating that vesicles of all size were elevated after injury. Treatment of EC with the plasma samples from patients tended to increase adhesion of peripheral blood mononuclear cells compared to samples from healthy controls; the increase was quite consistent over time but was not statistically significant at any one time.

There have been a number of studies of plasma EV following traumatic injury, but not over such a prolonged period, comparing different methods of quantitation, and attempting to correlate numerical analysis to pro-inflammatory function. Others have shown elevation of EV soon after trauma, assessed by FC [6,16], and also demonstrated pro-coagulant activity associated with vesicles [7, 25]. Curry *et al*. [7] reported increased numbers of EV derived from platelets and red cells, but not from leukocytes or EC. Our study did detect increase in EV from EC and from leukocytes, albeit from a very low baseline for the latter. Levels of EV were not correlated with the circulating blood counts for the parent cells, and so changes were not due to changes in these numbers after traumatic injury. Our results thus suggest ongoing activation and/or damage to endothelium, platelets and leukocytes within the vasculature, likely because of continuing elevation of inflammatory cytokines and thrombotic mediators, directly induced by the injury and/or driven by the following acute phase response.

NTA has been proposed as a sensitive method for enumerating EV in biological fluids over a much wider range of size than FC [33]. We measured EV counts ~10^11^/ml in plasma by NTA, compared to ~10^6^−10^7^/ml by FC. The flow cytometer count will depend on the sensitivity of the particular device to detection of smaller vesicles. Here we were able to set a counting window based on polystyrene beads, which defined an approximate detectable range of diameters of 500–1000μm. Analysis of size distributions of samples by NTA indicated that <1% were of diameter >500nm while 1–2% were >400nm, suggesting that flow cytometer counts should have been higher than we measured. This may suggest that the flow cytometer did not detect all particles even within the largest size range. However, the FC calibrating beads have higher refractive index than microvesicles, so that the actual sizes detected may have been higher than predicted [37,38]. In addition, the total NTA count may be influenced by lipoproteins and chylomicrons which may be sensed. NTA has not been widely used in clinical studies to date. There was general agreement with FC that numbers of EV were elevated after trauma. Interestingly, while total NTA counts did not correlate with flow cytometer counts on a sample by sample basis, the NTA counts above 400nm did correlate with the flow cytometer counts. This suggests that either device could quantify this population, and supports the conclusion that they were elevated after traumatic injury. Although the flow cytometer was restricted to the larger EV, it did allow analysis of their source. Analysis of fluorescent labelled EV is possibly using more advanced NTA systems (e.g. NS500 system, Malvern Instruments), but this remains problematic in relation to sensitivity for the smaller vesicles [39]. Our results thus demonstrate the desirability of using both techniques to analyse EV numbers and sources in comparisons between healthy and pathological states.

Platelet-derived EV have been shown to have a high procoagulant activity [40] and to be associated with a range of thrombotic disorders including traumatic injury [7, 25, 40, 41]. The abundance of platelet EV in plasma has been shown to vary widely even in healthy donors [15, 16, 42], and it has been suggested that a proportion of circulating EV that are attributed to platelets may actually be derived from megakaryocytes [17, 43]. We included two putative surface markers for platelets because of the importance given to platelet EV in previous studies of circulatory pathology, and the desire to assess which might be the more appropriate in the context of the present study. We found similar numbers overall of EV that were CD41^+^ or CD42b^+^, but a weak correlation between the two on a sample by sample basis. CD41+ gave better discrimination between patients and controls, with a lower variability between samples and greater discrimination between e.g. day1 trauma and controls (ratios of medians were 4.5 vs. 2.5 for CD41^+^ vs. CD42b^+^ EV). These results suggest that the two types of EV may not all have been from same source, i.e., activated platelets. GpIb is known to be shed from activated platelets [44], but in soluble form would not be detected on EV. It is possible therefore that increase in numbers of CD41+ EV was a more effective marker of platelet activation here, showing better discrimination above an underlying number of EV arising from megakaryocytes as well as platelets. Previous studies have tested various platelet markers that define the counts and functional effects of platelet derived vesicles [45]. In the light of above uncertainties about the best assay for their release, we also tested pro-coagulant activity in samples, which was indeed found to correlate with EV count by flow cytometry.

It is also interesting that analysis of different types of EV with specific markers gave better discrimination between controls and trauma patients than the total EV count by flow cytometry. The difference may arise from the unchanging presence of an underlying number of EV not attributable to the cell types tested and also to noise arising in the light scatter detection used for the total count. Fluorescence detection is less prone to this non-specific background.

Traumatic injury may lead to the development of an intense systemic inflammatory response syndrome involving activation of the innate immune system, including inflammatory cell migration [46, 47]. Increased levels of plasma EV may play a role in inflammatory processes in complications such as sepsis and multiple organ dysfunction syndrome [20, 21], and increased platelet EV count was associated with sepsis following trauma [6]. On the other hand, lower platelet EV count was associated with impaired coagulation [16], and among trauma patients, those who died had fewer CD41+ EV than those who survived [7]. The current study was not powered to consider the links between EV number or type and clinical complications or outcome. However, it seems that in addition to promoting deleterious inflammation, the pro-coagulant effects of EV might be useful under some circumstances. Thus, more detailed studies of how changes in count and function are linked to pathophysiology and outcome are required to resolve these issues.

Mechanistic studies in vitro showed that platelet EV acted on EC to upregulate ICAM-1 [48] and transferred chemokines [13], thus increasing leukocyte adhesion. Leukocyte EV may also interact with and activate EC [49]. We thus investigated whether patient-derived plasma contining EV could directly promote leukocyte adhesion if incubated with EC for 24h, or potentiate their response to low dose TNF. Overall, the samples prepared from patients who had suffered traumatic injury induced a higher level of adhesion of mononuclear cells than samples from healthy controls, but did not increase neutrophil adhesion. Effects were again prolonged after trauma. The effect on monocyte adhesion was greater for the otherwise unstimulated EC than those treated with TNF. Analysis of serum from patients in the Golden Hour study, indicate that there is elevation of several cytokines (IL-6, TNF and IL-8) within the first day after trauma [50]. Observations in the SIRS study also show elevation in cytokines in the days after injury [51]. Given the modest functional effect we observed and the paucity of material available, we did not attempt to separate effects of EV and cytokines by removing EV from samples and re-testing their effect on endothelial cells. Moreover, the cytokine levels values varied widely between patients, as did the EV count measured here, and so their separate effects would unlikely be distinguishable in the samples studied here. Thus, further studies would be needed, e.g., on a chosen cohort with stronger effects on endothelial responses, and perhaps higher EV and cytokine levels, where one could test whether separate responses could be attributed to EV and/or soluble mediators.

A problem underlying detailed analysis of EV, their function, role in pathogenesis and utility as biomarkers in this study, is the great heterogeneity in the patient population studied and their treatments. Patients received a range of variable treatments, including infusion of plasma expanders or blood, with uncertain effects on EV count. It is interesting that patterns could be discerned in the patients, but a very large study would be needed to dissect the effects e.g., of treatment variables, and test whether EV had prognostic value on such a background. In addition, the current study used samples which were subsets from two larger clinical studies, GHS and SIRS. Due to restriction in volume available, samples from the former were only used for the NTA analysis (total EV load) which we considered the primary parameter here. Both showed similar trends of prolonged elevation of EV count after injury, although values were higher in the GHS study. The reason for this is unclear as the methodology was the same, except that GHS samples were held at room temperature before analysis and SIRS were held on ice. Pre-analytical procedures are recognised to potentially affect EV levels [29–32], with counts tending to rise gradually for samples stored at room temperature, and cold causing changes in platelet morphology that have not been directly linked to vesicle shedding. Variations in procedure will add noise to the analysis, but do not in this case explain the greater number of EV detected post-trauma compared to healthy controls which were treated the same as the GHS samples. It is also notable that the SIRS samples showed elevation in subsets of EV from different cellular sources judged by flow cytometry, and not just from platelets which may be sensitive to cold storage. Thus, while there were widely varying results between EV counts, the elevation induced by trauma was not attributable to biological variation rather than methodological problems.

In summary, traumatic injury is associated with elevation of circulating EV that is prolonged and attributable to activation or damage to cells of the blood and vessel wall. Analysis of larger EV by FC and of a wider range of EV by NTA were complementary and showed similar trends. Plasma EV from patients showed pro-coagulant activity, and modest effects on endothelial responses tested separately in flow assays for the first time. However, the counting methodology of either type gave very large variation between donors. Indeed, there are several sources of variation, some methodological and some linked to treatment of patients, in addition to the effects of the trauma per se. This suggests that studies aimed at testing the association between EV and the epidemiology, patterns, and causes of complications following trauma will need to be on a large scale, to discriminate useful information from confounding factors. Counting methods may need to be combined with actual functional effects of EV. Here we tested effects on leukocyte adhesion to endothelial cells to see if that could yield a clear 'inflammatory' indicator. The results were again variable, and it would be simpler and perhaps more reproducible to measure other indicators for outcome linked to inflammation, such as circulating cytokines. We recently reported such measures as part of the Golden Hour study, where great inter-donor variation was again evident, but there was clear evidence of an immediate and prolonged inflammatory response [50]. The PPL assay used here is a 'routine' assay which showed good discrimination between patients and controls, and likely reflects the procoagulant effect of the major EV population (i.e., detected by NTA). It is relevant to thrombotic problems post injury, and we conclude that a functional assay of this type would at least complement counting of EV in future larger studies.

## Supporting information

S1 Fig The frequency distribution of EV diameter derived by NTA in plasma from controls and patients at different times after injury. Data are for controls, GHS 1 hour (T0), Day 1 (pooled GHS and SIRS), Day 3 (pooled GHS and SIRS) and subsequent days (SIRS only). Data are median values.
(TIF)

S1 File Data for all studies. NTA, FC and leukocyte adhesion data tabulated for controls and patients.
(PDF)

## Abbreviations

EC: Endothelial cells
EV: Extracellular vesicles
FC: Flow cytometry
HUVEC: Human umbilical vein endothelial cells
NTA: Nanoparticle tracking analysis
PBMC: Peripheral blood mononuclear cells
TNF: Tumour necrosis factor-α
