# Supplementary figures and images for: Changes in the pattern of plasma extracellular vesicles after severe trauma
Source: PLoS One. 2017 Aug 24;12(8):e0183640. doi: 10.1371/journal.pone.0183640 (PMC5570308; doi:10.1371/journal.pone.0183640)

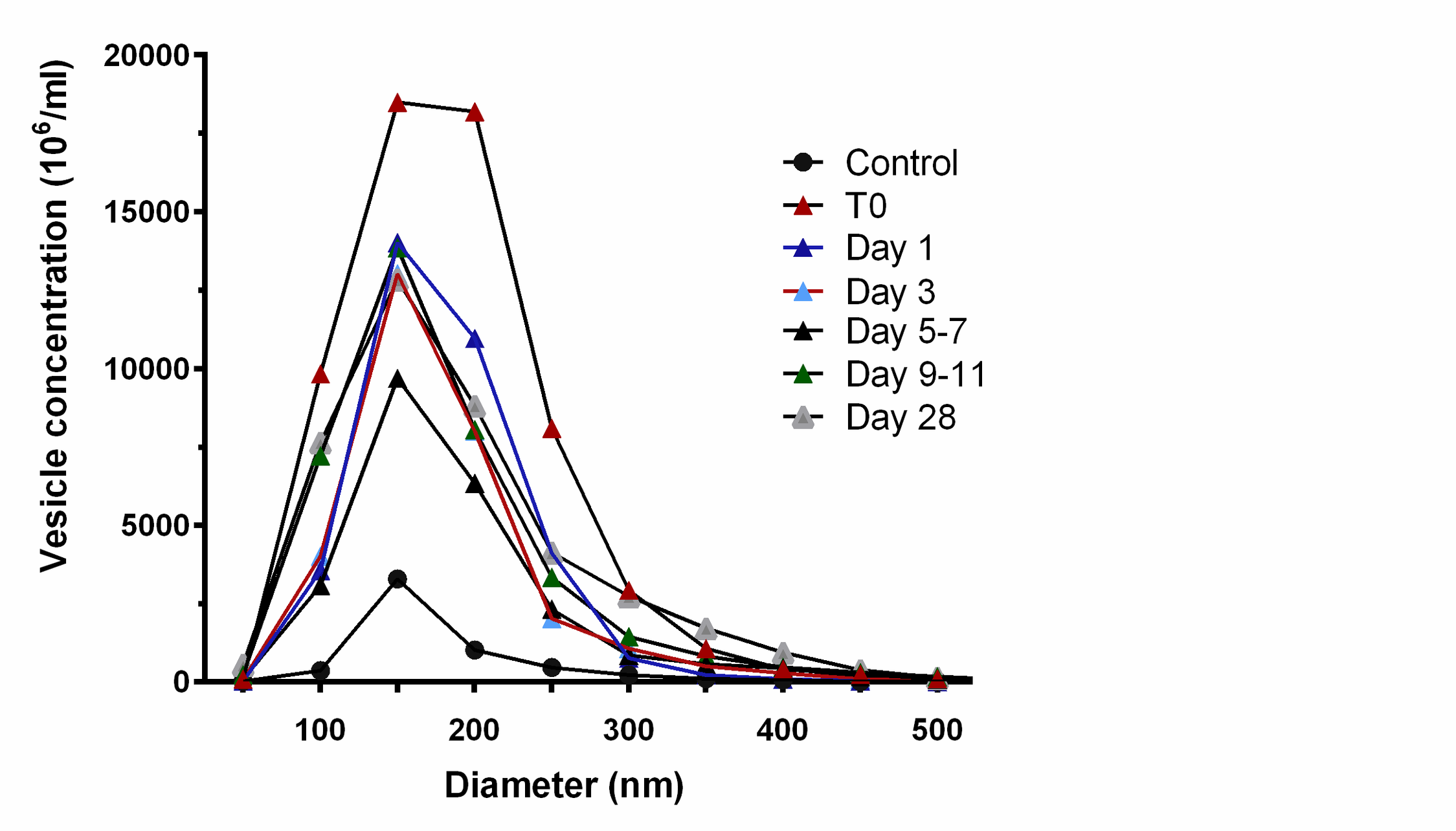

Supplement: S1 Fig — Data are for controls, GHS 1 hour (T0), Day 1 (pooled GHS and SIRS), Day 3 (pooled GHS and SIRS) and subsequent days (SIRS only). Data are median values. (TIF) [file pone.0183640.s001.tif]
