# Supplementary material for: Changes in the pattern of plasma extracellular vesicles after severe trauma
Source: PLoS One. 2017 Aug 24;12(8):e0183640. doi: 10.1371/journal.pone.0183640 (PMC5570308; doi:10.1371/journal.pone.0183640)
Supplement: S1 File — NTA, FC and leukocyte adhesion data tabulated for controls and patients. (PDF) [file pone.0183640.s002.pdf]

Nanoparticle tracking analysis

Total particle count/ml

Controls	SIRS	Day 1	Day3	Day5-7	Day9-11	Day28	Golden Hour	T0 (1h)	Day1	Day3
6.00E+09	SIR085	1.33E+10	1.59E+10	5.87E+09	1.91E+10		GH003	6.99E+10	1.25E+11	4.33E+10
2.16E+10	SIR086	4.38E+10	5.53E+10		1.78E+11		GH005	3.28E+10	6.32E+10	2.00E+11
1.85E+10	SIR091	2.96E+10			3.40E+10	5.87E+10	GH016	1.10E+11	1.16E+11	4.28E+10
5.65E+09	SIR092	5.71E+10	5.09E+10			6.35E+10	GH021	1.71E+11	3.97E+10	1.63E+11
7.00E+09	SIR095	2.23E+10	1.14E+10			4.02E+10	GH025	1.50E+11	6.17E+10	8.89E+10
2.60E+10	SIR096	4.65E+10		4.94E+10	6.71E+10		GH036	2.69E+11	2.07E+11	3.29E+10
2.85E+09	SIR098	1.33E+10	1.63E+10	5.87E+09	1.91E+10		GH045	1.02E+10	3.68E+10	4.04E+10
4.65E+09	SIR099	3.61E+09	1.01E+11		9.19E+09	1.50E+10	GH066	4.43E+10	2.17E+10	6.85E+10
1.65E+09	SIR100	1.24E+11	1.20E+12		3.63E+11	4.04E+11	GH074	9.67E+10	6.84E+10	3.29E+10
1.19E+10	SIR093	1.22E+09			2.21E+10	7.32E+09	GH093	2.19E+12	2.77E+12	6.15E+11
1.54E+09	SIRens025	2.28E+09	1.09E+09		9.03E+09	2.12E+10	GH128	6.79E+09	4.03E+09	1.54E+10
2.01E+09	SIRens030	2.15E+09	2.54E+10	2.58E+10	6.90E+08		GH149	7.89E+09	2.16E+10	5.17E+09
2.30E+09	SIRens024	1.14E+10	1.08E+09	1.22E+09			GH196	4.61E+10	1.92E+10	1.09E+10
7.67E+09	SIRens031	1.54E+10		8.43E+09	9.48E+09	2.62E+10	GH217	5.05E+10	8.46E+10	7.21E+09
5.20E+09	SIRens028	4.77E+11			2.35E+10		GH236	1.25E+11	8.68E+10	9.41E+10
2.01E+10	SIRens026	6.30E+08	6.05E+08	1.49E+09			GH243	3.57E+10	3.89E+10	4.14E+10
3.87E+10	SIRens022	3.45E+10		5.53E+09	4.13E+10	6.99E+10	GH285	3.02E+11	7.14E+10	3.07E+11
3.56E+10	SIRens029	1.18E+09		3.00E+10			GH329	5.18E+10	6.88E+10	1.16E+10
1.30E+09										
2.07E+09										
1.09E+10										
6.04E+09										
5.73E+09										
2.78E+09										

Flow Cytometry

Total particle count/ml

Controls	SIRS	Day 1	Day3	Day5-7	Day9-11	Day28
1.96E+06	SIR085	4.51E+05	6.31E+05		5.46E+05	
2.37E+05	SIR086	1.45E+06	1.86E+06		2.17E+07	5.88E+06
7.38E+05	SIR091	7.82E+06	1.04E+07		3.92E+06	2.78E+07
2.55E+05	SIR092	1.87E+06	4.53E+06	6.62E+06	2.08E+06	5.56E+07
4.24E+05	SIR095	1.46E+07	2.23E+07	1.51E+07	1.89E+08	7.92E+06
8.33E+05	SIR096	1.41E+07	5.01E+07	7.48E+05	7.42E+07	
1.35E+06	SIR098	5.48E+07	1.95E+07		7.38E+05	
3.12E+06	SIR099	1.25E+06	1.49E+07		1.77E+06	5.60E+06
	SIR100	1.64E+06	7.16E+06		2.63E+06	4.64E+06
	SIR093	9.50E+05	2.31E+05		5.82E+05	1.10E+06
	SIRens025	8.28E+05		1.47E+06		2.33E+06
	SIRens022	1.03E+06	1.95E+06	4.56E+06	1.90E+07	1.90E+07
	SIRens029	1.37E+06	1.82E+06	1.96E+06	2.71E+06	
	SIRens026	2.94E+06	3.19E+05	2.64E+05	1.62E+05	2.95E+05
	SIRens030	3.62E+06	5.07E+06	2.76E+06	3.09E+05	3.74E+05
	SIRens031	4.20E+05	5.89E+05			8.01E+05
	SIRens024	2.98E+05	2.96E+05	4.25E+05		

Flow Cytometry-Particle count/ml

CD41 positive

Controls	SIRS	Day 1	Day3	Day5-7	Day9-11	Day28
6.44E+04	SIR086	1.53E+05	4.00E+05			7.08E+05
1.25E+05	SIR091	9.90E+04	2.30E+05			
6.38E+04	SIR092	3.56E+05	6.01E+05		2.31E+05	
2.18E+04	SIR095	2.02E+05	1.41E+05			2.30E+05
1.33E+04	SIR096	3.64E+05		1.03E+06		
4.21E+04	SIR098	3.38E+05	1.80E+05	3.58E+05	2.28E+05	
7.88E+04	SIR099	2.19E+05	3.83E+05		3.63E+05	3.34E+05
6.76E+04	SIR100	6.43E+05	4.50E+05		2.65E+05	2.26E+05
7.10E+04	SIR093	1.94E+05	2.21E+05		2.16E+05	3.12E+05
5.89E+04	SIRens025	2.53E+05	1.89E+05			4.43E+05
9.14E+04	SIRens022	6.68E+05		7.42E+05		
	SIRens026	2.78E+05	2.64E+05	2.55E+05	2.29E+05	
	SIRens030	2.27E+05	2.75E+05	2.51E+05		
	SIRens024	1.83E+05	6.13E+04	2.71E+05	1.64E+05	2.04E+05
	SIRens031	1.17E+06		1.66E+05		4.49E+05
	SIRens029	2.91E+05	2.55E+05			

Flow Cytometry-Particle count/ml

CD42 positive

Controls	SIRS	Day 1	Day3	Day5-7	Day9-11	Day28
1.38E+06	SIR086	5.15E+04	5.67E+04			1.01E+06
1.21E+05	SIR091	1.73E+05	2.66E+05			1.02E+06
3.57E+04	SIR092	2.10E+05	8.75E+05		2.05E+05	
1.28E+05	SIR095	4.48E+04	1.63E+05			3.67E+06
9.61E+04	SIR096	3.14E+05		5.15E+05		
5.56E+04	SIR098	1.14E+05	1.73E+05	8.43E+04	2.28E+05	
1.12E+04	SIR099	1.73E+05	1.29E+06		5.05E+05	1.56E+06
6.10E+04	SIR100	2.46E+05	1.07E+06		9.52E+04	1.31E+05
8.30E+04	SIR093	1.34E+05	2.62E+05		1.11E+05	3.74E+05
5.15E+04	SIRens025	1.13E+05	4.61E+04			7.67E+05
1.14E+04	SIRens022	2.13E+05		1.25E+06		
	SIRens026	7.93E+04	8.48E+04	8.64E+04	6.26E+04	
	SIRens030	6.62E+04	7.71E+04	6.31E+04		
	SIRens024	3.62E+05	2.06E+05	2.00E+05	9.91E+04	9.61E+04
	SIRens031	4.88E+05		9.39E+04		1.21E+06
	SIRens029	1.04E+05	1.30E+05			

Flow Cytometry-Particle count/ml

CD144 positive

Controls	SIRS	Day 1	Day3	Day5-7	Day9-11	Day28
1.01E+06	SIR085	3.15E+05	3.75E+05		6.48E+05	
1.58E+05	SIR086	1.18E+06	1.36E+06		1.88E+07	
1.68E+05	SIR091	4.47E+06			2.09E+06	6.90E+06
1.84E+05	SIR092	2.05E+05	1.06E+06	3.76E+05		1.06E+06
1.17E+05	SIR095	1.11E+07	9.39E+06			
1.40E+05	SIR096	9.25E+06	9.13E+04		2.68E+07	
6.75E+04	SIR098	4.08E+07				
1.79E+05	SIR099	5.99E+05	3.77E+05		1.01E+06	9.06E+06
	SIR100	1.95E+06	1.67E+07		2.72E+06	2.90E+06
	SIR093					
	SIRens025	3.71E+05		2.05E+05		1.61E+06
	SIRens022	5.96E+05	5.85E+05	1.26E+06		5.04E+06
	SIRens026	1.02E+06	1.70E+06	1.76E+06		
	SIRens030	5.30E+06	1.64E+05	5.68E+04	7.00E+04	
	SIRens024	2.53E+06	2.53E+06	2.08E+06		
	SIRens031	4.77E+04			7.77E+04	6.57E+04

Flow Cytometry-Particle count/ml

CD45 positive

Controls	SIRS	Day 1	Day3	Day5-7	Day9-11	Day28
3.76E+03	SIR085	1.73E+04	6.77E+03		3.61E+03	
1.05E+03	SIR086	6.43E+03	1.73E+04		1.49E+06	
2.37E+03	SIR091	1.31E+05			1.53E+05	1.97E+06
9.11E+03	SIR092	3.52E+04	4.00E+04	2.90E+04		4.00E+04
1.13E+03	SIR095	3.42E+05	2.20E+05		1.61E+07	
2.32E+03	SIR096	3.67E+05	5.42E+05		3.80E+05	
2.57E+03	SIR098	8.25E+05				
1.42E+03	SIR099	5.42E+03	1.21E+04		3.10E+04	2.48E+05
	SIR100	9.64E+04	3.29E+05		1.10E+05	7.48E+06
	SIRens025	1.98E+03		1.52E+03		4.08E+04
	SIRens022	6.23E+05	1.15E+05	1.25E+05	7.43E+05	7.43E+05
	SIRens026	1.65E+04	2.93E+04	3.63E+04	1.26E+05	
	SIRens030	3.04E+05	3.49E+03	2.71E+03	1.53E+03	
	SIRens024		3.16E+04			
	SIRens031	2.08E+04			1.29E+04	8.82E+03

	PBMC Adhesion to unstimulated EC - Relative to control				PBMC Adhesion to TNF-treated EC - relative to control			
	Day1	Day3-5	Day9-11	Day28	Day1	Day3-5	Day9-11	Day28
SIR093	0.902	1.083	1.568	1.516	0.902	1.083	1.568	1.516
SIR100	4.722	3.292	3.333	7.208	2.036	1.185	1.695	0.913
SIR092	2.374	1.077	0.692	0.862	0.995	1.355	2.123	2.497
SIR099	0.864	1.335	0.407	1.653	2.411	0.760	1.661	1.136
SIRens029	0.909	1.195		1.039	0.574	0.766	0.894	0.620
SIRens031	1.200	1.000	0.960	0.900	0.711	1.219	0.810	1.057
SIRens022	6.000	9.917	4.667	7.500			1.182	
SIRens028	0.062		0.375		1.403	1.973	1.684	
SIR086	3.176	2.353	4.529		1.267	0.935	1.111	
SIRens030	0.615	0.176	0.879		0.900	0.932	1.251	
SIR091	1.438	1.630	1.215		1.187	1.013	0.949	
SIR098	1.000	1.550			0.645	0.436		
SIR095	1.091	0.606		0.364	1.425	0.883		1.369
SIR094	0.681	0.731			0.783	0.999	0.899	
SIRens024	0.762	1.349	1.381		2.250	1.639		
SIRens026	0.278	0.694	0.278		1.109	1.375	1.000	
	Control				0.944	1.230	1.771	

	Neutrophil Adhesion to unstimulated EC - Relative to control				Neutrophil Adhesion to TNF-treated EC - relative to control			
	Day1	Day3-5	Day9-11	Day28	Day1	Day3-5	Day9-11	Day28
SIR093	4.286	1.500	1.833	3.000	0.887	0.865	0.816	0.820
SIR100	1.204	1.139	1.898	1.889	2.325	0.628	2.000	1.944
SIR092	0.746	1.083	0.417	0.833		2.222	2.014	1.479
SIR099	0.218	0.143	0.303	1.795	1.385		1.577	1.631

Flow Cytometry-Particle count/ml

CD42	CD41
9.61E+04	1.33E+04
1.28E+05	2.18E+04
5.56E+04	4.21E+04
5.15E+04	5.89E+04
2.06E+05	6.13E+04
3.57E+04	6.38E+04
1.38E+06	6.44E+04
6.10E+04	6.76E+04
8.30E+04	7.10E+04
1.12E+04	7.88E+04
1.14E+04	9.14E+04
1.73E+05	9.90E+04
1.21E+05	1.25E+05
1.63E+05	1.41E+05
5.15E+04	1.53E+05
9.91E+04	1.64E+05
9.39E+04	1.66E+05
1.73E+05	1.80E+05
3.62E+05	1.83E+05
4.61E+04	1.89E+05
1.34E+05	1.94E+05
4.48E+04	2.02E+05
9.61E+04	2.04E+05
1.11E+05	2.16E+05
1.73E+05	2.19E+05
2.62E+05	2.21E+05
1.31E+05	2.26E+05
6.62E+04	2.27E+05
2.28E+05	2.28E+05
6.26E+04	2.29E+05

Correlation CD42 vs. CD41 positive

CD42	CD41
2.66E+05	2.30E+05
2.05E+05	2.31E+05
6.31E+04	2.51E+05
1.13E+05	2.53E+05
1.30E+05	2.55E+05
8.64E+04	2.55E+05
8.48E+04	2.64E+05
9.52E+04	2.65E+05
2.00E+05	2.71E+05
7.71E+04	2.75E+05
7.93E+04	2.78E+05
1.04E+05	2.91E+05
3.74E+05	3.12E+05
1.56E+06	3.34E+05
1.14E+05	3.38E+05
2.10E+05	3.56E+05
8.43E+04	3.58E+05
5.05E+05	3.63E+05
3.14E+05	3.64E+05
1.29E+06	3.83E+05
5.67E+04	4.00E+05
7.67E+05	4.43E+05
1.21E+06	4.49E+05
1.07E+06	4.50E+05
8.75E+05	6.01E+05
2.46E+05	6.43E+05
2.13E+05	6.68E+05
1.01E+06	7.08E+05
1.25E+06	7.42E+05
5.15E+05	1.03E+06
4.88E+05	1.17E+06
